# Majority of New Onset of Dental Caries Occurred from Caries-Free Students: A Longitudinal Study in Primary School Students

**DOI:** 10.3390/ijerph17228476

**Published:** 2020-11-16

**Authors:** Taro Kusama, Hidemi Todoriki, Ken Osaka, Jun Aida

**Affiliations:** 1 Department of International and Community Oral Health, Tohoku University Graduate School of Dentistry, Sendai 980-8575, Japan; kusama-thk@umin.ac.jp (T.K.); ken.osaka.e5@tohoku.ac.jp (K.O.); 2 Tropical Biosphere Research Center, University of the Ryukyus, Okinawa 903-0213, Japan; todoriki@comb.u-ryukyu.ac.jp; 3 Department of Oral Health Promotion, Graduate School of Medical and Dental Sciences, Tokyo Medical and Dental University, Bunkyo-ku, Tokyo 113-8549, Japan; 4 Division for Regional Community Development, Tohoku University Graduate School of Dentistry, Sendai 980-8575, Japan

**Keywords:** dental caries, prevention paradox, universal health coverage, population approach, longitudinal study

## Abstract

We examined Rose’s axiom that a large number of people exposed to a small risk may generate more cases than a small number exposed to a high risk, using data on caries incidence. This longitudinal study was based on the records of annual dental checks conducted in primary schools in Okinawa, Japan. Participants were students aged 6–11 years at baseline in 2014, and a follow-up survey was conducted after one-year. The outcome variable was the increased number of decayed, missing, and filled teeth (DMFT). The predictor variable was the baseline DMFT score. Gender, grade, and affiliated school variables were adjusted. A negative binomial regression model was used to obtain the estimated increase of DMFT score. Among 1542 students, 1138 (73.8%) were caries-free at baseline. A total of 317 (20.6%) developed new caries during the follow-up. The predicted number of new carious teeth in a caries-free students and students with DMFT = 1 at baseline were 0.26 (95% CI, 0.22–0.31) and 0.45 teeth (95% CI, 0.33–0.56), respectively. However, among the total of 502 newly onset of carious teeth, 300 teeth (59.7%) occurred from the caries-free students at baseline. Hence, prevention strategies should target the low-risk group because they comprise the majority of the population.

## 1. Introduction

Dental caries is the most prevalent disease around the world, affecting 2.4 billion people [1]. The prevalence of dental caries has remained approximately stable from 1990 to the present day [2]. Caries and other oral diseases cause considerable indirect impacts in the form of financial burdens [3,4], loss of work productivity [5,6], and deterioration of quality of health [7]. Universal health coverage (UHC) and prevention and treatment of non-communicable diseases (NCDs) are key targets in the Sustainable Development Goals (SDGs) by 2030. The importance of oral health in the context of UHC and NCDs has been recognized [8]. The UHC ensures essential health services for everyone; therefore, it is necessary to formulate health strategies, which would be available to all [9,10]. As a part of the UHC, focus is required not only the treatment but also the prevention of caries to address the rise in incidence [10]. However, currently oral health care emphasizes more on treatments rather than prevention [11]. Hence, to tackle oral diseases, a downstream intervention which is oriented to treatment is not sufficient, and upstream intervention which includes comprehensive prevention system is needed in UHC framework [11,12].

Rose pointed out one of the most fundamental axioms in preventive medicine that “a large number of people exposed to a small risk may generate many more cases than a small number exposed to a high risk” [13]. Therefore, focusing on high-risk individuals alone ignores the majority of new incidents of disease. This causes a “prevention paradox” that effective prevention measures for communities have small effects for each individual, as individuals have small risks [13]. The axiom in preventive medicine supports the importance of “population approach” that a preventive intervention aims to affect all populations. The similarity between the population approach and UHC is the availability of the intervention to all populations. However, in the dental field, studies on the distribution of disease risk are scarce, although the distributions of wider health risks have been examined in the other medical fields [14], and the concept has still been attracting attention [15]. Only one descriptive study [16] and one cross-sectional study [17] have been carried out and there is no elucidation by longitudinal design analysis with a statistical model. Therefore, the importance of prevention for the caries-free population (low-risk population) was not emphasized greatly in previous studies. We hypothesized that the majority of new-onset of caries occurred from caries-free students. If this is true, it is considered that the prevention paradox exists in dental caries, which directly relates to prevention policies. The present study targeted primary school students because they are in the susceptible stage for caries incidence because of permanent teeth eruption and effective prevention strategies are required [18,19]. Thus, we used a longitudinal design to examine whether most of the new-onset caries occurred from the caries-free population among primary school students and discuss the importance of population prevention strategies in the UHC.

## 2. Materials and Methods

### 2.1. Settings and Participants

This longitudinal study was based on data from annual school dental health examinations in primary schools. The data were obtained through a food education program in Yaese town, Okinawa prefecture, Japan [20]. Dental examinations were conducted by school dentists who checked for the presence of decayed, missing, and filled teeth (DMFT) for each permanent tooth. The diagnostic criteria for dental caries was based on the “Oral health surveys: basic methods-5th edition” [21]. The data regarding oral condition was secondarily obtained from annual school dental checkups determined by law, and we could not conduct calibration. However, the same dentist examined all students in each school at baseline and follow-up surveys. The eligibility of participants was students in the first to the fifth grade, aged 6 to 11 years at baseline, and we included all students in the analysis unless failed to follow up. The survey was conducted in four primary schools. The baseline survey was conducted in June 2014, and a one-year follow-up survey was conducted in June 2015. 

### 2.2. Outcome Variable

We used the increase in DMFT score and the incidence of new onset caries one year after baseline as outcome variables. We subtracted the DMFT score in 2014 from that in 2015 to calculate the increase. In our analysis, the caries increase of 158 students was negative. However, the range of negative increase was within the baseline DMFT score. Therefore, it is considered that filled-teeth by composite resin were misclassified as sound teeth because of their white color. For this reason, we treated the negative value in the outcome variable as zero. The increased DMFT score was also dichotomized to create a caries incidence variable. If the DMFT score increased by one or more, it was regarded as a case of new onset caries.

### 2.3. Predictor Variable and Covariates

We used the DMFT score at baseline as the predictor variable. A previous study indicated that those who have experienced caries are at a higher risk of developing new caries compared to those who were caries-free [18]. We used gender, grade at baseline, and affiliated primary school as covariates.

### 2.4. Statistical Analysis

For the main analysis, we used the increase in DMFT score as the outcome. We estimated the relative risks (RRs) and 95% confidence intervals (CIs) of the increase in DMFT scores using a negative binomial regression model with a Huber–White sandwich estimator for standard errors. Based on the negative binomial model, we calculated the estimated number of newly onset carious teeth per student after one year by each category of DMFT at baseline. To estimate the total number of new onset carious teeth, we multiplied the estimates by the distribution of DMFT at baseline and predicted the sum of the numbers of the new carious teeth from each DMFT category at baseline. For a sensitivity analysis using caries incidence as the outcome, we estimated the RRs and 95%CIs of caries incidence using a Poisson regression model with a Huber–White sandwich estimator for standard errors to predict the absolute risk of caries incidence. We also estimated the total number of caries cases from each DMFT category at baseline. We did not conduct statistical imputation to avoiding selection bias for the missing value in the outcome and treated them as drop-outs because the follow-up rate was nearly 100%. Stata/MP version 15 (Stata Corp., College Station, TX, USA) was used to perform statistical analysis.

### 2.5. Ethical Statement

We explained the survey to the guardians of all participants and obtained written informed consent from the children and the guardians themselves. The study was approved by the Ethics Committee for Epidemiological Research at the University of the Ryukyu (approval no. 216, approval date 31 January 2014). We followed the STROBE protocol to present this study.

## 3. Results

**Figure 1:**
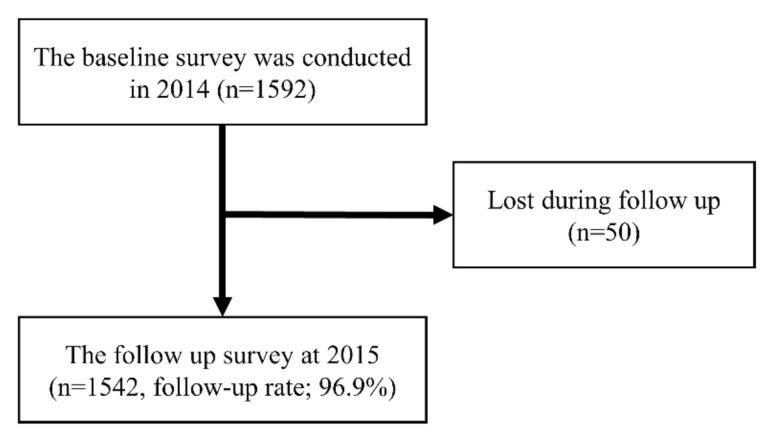
The participants flow for analytic sample (*n* = 1542).

**Figure 2:**
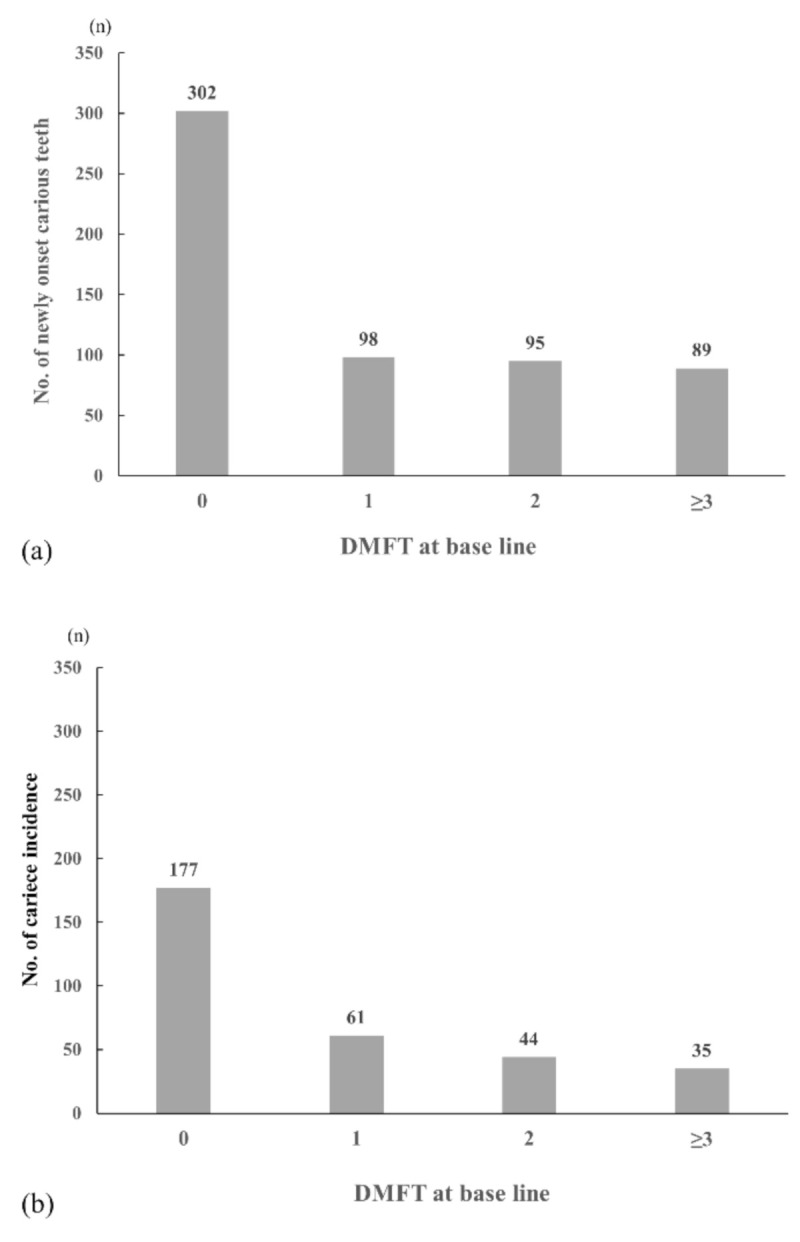
(**a**) Number of newly onset carious teeth at the one-year follow-up by DMFT at baseline. Most of the carious teeth occurred in students previously caries-free at baseline. (**b**) Number of participants with caries incidence during one-year follow-up by DMFT at baseline. MDFT: decayed, missing, and filled teeth.

For the baseline survey in 2014, a total of 1592 students were examined. For the follow-up survey in 2015, 1542 students participated in a second dental examinations; the follow-up rate was 96.9%, and all those students were included in the analysis (Figure 1). Table 1 provides the descriptive characteristics of the participants. A total of 50.6% were male, and almost the same number of students was included from each school grade. There were 1138 (73.8%) caries-free students at baseline. The proportion of those who had experienced caries at the one-year follow-up was 20.6% (*n* = 317), and the mean number of new onset carious teeth was 0.38 (1 SD = 0.95). Figure 2a shows the distribution of the number of new onset carious teeth by DMFT at baseline. A total of 584 carious teeth were observed at follow-up, of which 302 occurred in participants who were caries-free at baseline. The participants who were caries-free at baseline occupied 51.7% of the total number of new onset caries. When we calculated the caries incidence in individual students rather than in teeth, the same result was observed (Figure 2b). Among 317 students who experienced new onset caries, 55.8% were caries-free at baseline. 

**Figure 3:**
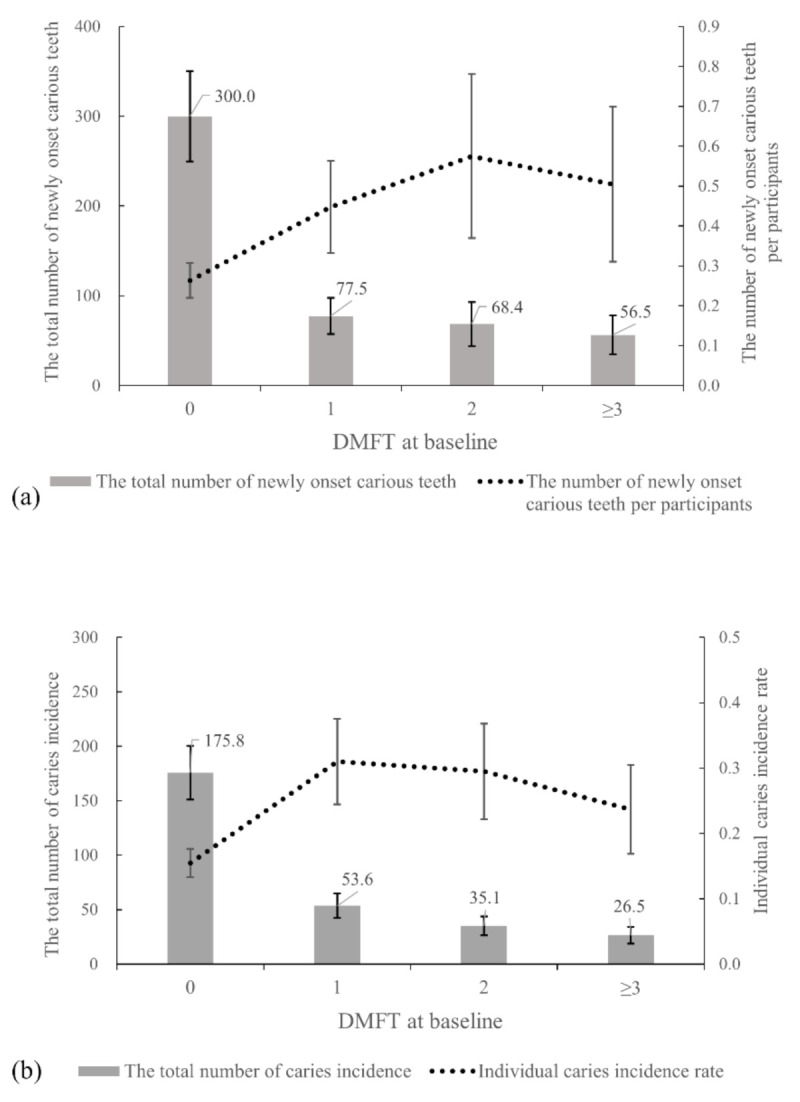
(**a**) The number of newly onset carious teeth per participant and the total number of carious teeth by DMFT at baseline. As DMFT at baseline rises, the number of newly onset carious teeth increases. However, the total number of newly onset caries is highest in the caries-free population (DMFT = 0) at baseline. (**b**) Individual caries incidence and the total number of students experiencing caries incidence by DMFT at baseline. Similar to the number of newly onset carious teeth, when DMFT ≥ 1 at baseline, the individual caries incidence rate is higher than DMFT = 0. However, the total number of individual caries incidence is highest in caries-free (DMFT = 0) at baseline.

To estimate the number of new onset carious teeth and individual incidences, we built a negative binomial regression model (Figure 3a and Supplementary Table S1). Although those with caries at baseline had a higher risk of caries, most of the new onset carious teeth occurred in those who were caries-free at baseline. Significantly higher RRs were observed (Figure 3b and Supplementary Table S2). However, the total numbers of teeth and individuals with caries incidence were highest among those, who were caries-free at baseline (300.0 teeth; 95% CI, 249.8–350.1 and 175.8 students; 95% CI, 151.1–200.5) with 59.7% of newly onset carious teeth and 60.4% of students experiencing caries incidence.

## 4. Discussion

Approximately 60% of the caries incidences occurred from students who were caries-free at baseline. The risk of caries was higher among those who had experienced caries at baseline. However, as a whole, the total number of caries incidence was higher among students who were caries-free at baseline. Only a few previous studies have implied the existence of the fundamental axiom that the majority of diseases occur from large numbers in the low-risk populations rather than those in the small high-risk population in caries incidence. However, these reports were based on descriptive or cross-sectional studies [16,17]. Therefore, the longitudinal relationship remains unclear. This is the first study to confirm the axiom in caries incidence using longitudinal data.

The mechanism of “prevention paradox” in caries incidence could be explained as follows: First, the risk of caries incidence is not equal to zero even in those who have not experienced caries at all; low-risk does not mean zero risk. A previous study suggested that the increment of DMFT was observed not only among those who experienced caries in childhood but also among those who did not [20]. Although those who experienced more caries were at a higher risk [18,22], the risk for those who did not experience caries was not zero [23]. Second, the high risk population is smaller than the low risk population. In fact, the mean of DMFT distribution was biased towards zero in the population of the present study. In other countries, the distributions of DMFT and caries prevalence in children are shifted to zero [24,25], and the majority of children are at low risk. Therefore, in these countries, “prevention paradox” will also be observed.

In terms of public health implications, this study supports the importance of a “health for all” strategy, such as population approach and UHC. From the results of the present study, the majority of diseases are caused in large numbers in low-risk populations. Therefore, high-risk strategies, such as dental checkups and treatment recommendations, may not be effective for caries prevention and the decline of caries prevalence [26]. The present situation of oral health care, which is treatment oriented, is considered insufficient to tackle the huge burden of dental caries. From the results of the present study, prevention strategies, which can reach the whole population are essential for caries prevention; the population approach, which can include low-risk populations is superior to the high-risk strategy for caries prevention in this context. Currently, the population approach has been expanded to reduce health inequalities, and named “proportionate universalism” as a whole population intervention improves the health of the high-risk group more than that of the low-risk group [27]. Interventions such as community-level fluoride applications, including community water fluoridation, school-based fluoride mouth rinse, or school-based sealant program, which can tackle the upstream of caries incidence can be effective population approaches and proportionate universalism [28,29,30]. These measures to prevent caries should be included as part of the UHC framework. Furthermore, sugar consumption also contributes to caries incidence and other NCDs [31]. Recently, commercial determinants of health have attracted increasing attention [32]. Commercial activities of food and beverage companies affect our dietary habits and increase sugar consumption. To tackle these matters, introducing sugar taxation is considered to be effective [33,34]. Some countries have introduced and achieved the results [35]. Policymakers need to consider the introduction of sugar taxation to prevent NCDs, including caries [11]. 

As a limitation, in this study, we used only age as a time-variant confounder. There is a possibility that the time-variant confounders cause bias. In relation to time-invariant confounders, we did not include in the model because, in our analysis model, the time-invariant confounders affect both baseline and follow-up caries. We compared the caries experience between the baseline and follow-up for each student and examined the association of the baseline caries level. Therefore, the effects of time-invariant individual confounders can be cancel-outed. Another limitation is the generalizability of this study. Because it was conducted in an area with relatively higher caries prevalence in Japan, the generalizability of the present study seems to be limited. However, the present result is consistent with previous studies in the United States and the United Kingdom [16,17]. In addition, the phenomenon that the majority of incidence occurs from the low-risk populations is widely known in other diseases [14]. In relation to the mechanism of the distribution of disease risk in populations, if the proportion of caries-free students increased, this paradoxical association would be strengthened. Therefore, the results of this study are considered to be applicable to other populations. Additionally, information bias due to misclassification as mentioned in the methods section existed, and this may influence our results. However, the misclassification is considered to be due to treatment and not due to overlooking. Therefore, the influence is considered to be relatively small.

## 5. Conclusions

The present study revealed that the majority of caries incidence occurred from caries-free students in primary school. The result implied the existence of “prevention paradox” in caries incidence. We must implement population strategies, such as community-level fluoride application and sugar taxation, for caries prevention as part of the UHC framework.

## Figures and Tables

**Table 1:** Characteristics of participants (*n* = 1542). MDFT: decayed, missing, and filled teeth.

	*n*	%
Sex		
Male	781	50.6
Female	761	49.4
School grade at base line		
1st	304	19.7
2nd	318	20.6
3rd	291	18.9
4th	307	19.9
5th	322	20.9
DMFT at baseline (2014)		
0	1138	73.8
1	173	11.2
2	119	7.7
≥3	112	7.3

## Supplementary Materials

The following are available online at https://www.mdpi.com/1660-4601/17/22/8476/s1, Table S1: The association between DMFT at baseline and increment of newly onset carious teeth (n = 1542); Table S2: The association between DMFT at baseline and individual caries incidence (n = 1542).
