# Supplementary material for: Majority of New Onset of Dental Caries Occurred from Caries-Free Students: A Longitudinal Study in Primary School Students
Source: Int J Environ Res Public Health. 2020 Nov 16;17(22):8476. doi: 10.3390/ijerph17228476 (PMC7698044; doi:10.3390/ijerph17228476)
Supplement: Supplementary file 1 [file ijerph-17-08476-s001.pdf]

Table S1. The association between DMFT at baseline and increment of newly onset carious teeth (n = 1542).

DMFT at base line	No. of participants	aRR (95%CI)*	The number of newly onset carious teeth per participants (95%CI)	The total number of carious teeth (95%CI)	The proportion of the total (%)
0	1138	Ref.	0.26 (0.22-0.31)	300.0 (249.8-350.2)	59.7
1	173	1.70 (1.26-2.29)	0.45 (0.33-0.56)	77.5 (57.4-97.5)	15.4
2	119	2.18 (1.49-3.20)	0.57 (0.37-0.78)	68.4 (43.9-92.9)	13.6
≥3	112	1.91 (1.27-2.88)	0.50 (0.31-0.70)	56.5 (34.8-78.2)	11.3

Table S2. The association between DMFT at baseline and individual caries incidence (n = 1542).

DMFT at base line	No. of participants	aRR (95%CI)*	Individual caries incidence rate (95%CI)	The total number of caries incidence (95%CI)	The proportion of the total (%)
0	1138	Ref.	0.15 (0.13-0.18)	175.8 (151.1-200.5)	60.4
1	173	2.00 (1.57-2.56)	0.31 (0.24-0.37)	53.6 (42.3-64.9)	18.4
2	119	1.91 (1.45-2.51)	0.29 (0.22-0.37)	35.1 (26.4-43.8)	12.1
≥3	112	1.53 (1.13-2.09)	0.24 (0.17-0.30)	26.5 (18.9-34.1)	9.1

Note: *Adjusted for gender, school grade, and affiliated school; aRR = adjusted relative risk, 95%CI = 95% confidence interval.
